# Prevalence of Azole-Resistant *Aspergillus* Section *Fumigati* Strains Isolated from Romanian Vineyard Soil Samples

**DOI:** 10.3390/antibiotics12121695

**Published:** 2023-12-03

**Authors:** Horațiu Alexandru Colosi, Alina Mihaela Baciu, Carmen Costache, Razvan Vlad Opris, Radu Anghel Popp, Marcela Sabou, Ioana Alina Colosi

**Affiliations:** 1 Department of Medical Education, Division of Medical Informatics and Biostatistics, Iuliu Hațieganu University of Medicine and Pharmacy, 400349 Cluj-Napoca, Romania; hcolosi@umfcluj.ro (H.A.C.); icolosi@umfcluj.ro (I.A.C.); 2 Division of Microbiology, Iuliu Hațieganu University of Medicine and Pharmacy, 400349 Cluj-Napoca, Romania; 3 Division of Medical Genetics, Iuliu Hatieganu University of Medicine and Pharmacy, 400349 Cluj-Napoca, Romania; 4 Laboratoire de Parasitologie et Mycologie Médicale, Les Hôpitaux Universitaires de Strasbourg, 67000 Strasbourg, France; amsabou@unistra.fr; 5 Institut de Parasitologie et de Pathologie Tropicale, UR7292 Dynamique des Interactions hôte Pathogène, Fédération de Médecine Translationnelle, Université de Strasbourg, 67000 Strasbourg, France

**Keywords:** azole resistance, *Aspergillus* section *Fumigati*, environmental samples, cryptic *Aspergillus*, non-wild-type *Aspergillus udagawae*

## Abstract

The relationship between fungal species and their resistance patterns in vineyard soils has important implications for agriculture and medicine. This study explored the prevalence of *Aspergillus* section *Fumigati* species and their resistance to azole compounds in Romanian vineyard soils. Methods: A total of 265 soil samples from various Romanian vineyards were screened for fungi resistant to azoles. Results: *Aspergillus* section *Fumigati* isolates exhibited significant resistance to itraconazole and voriconazole, but no azole-resistant *Aspergillus fumigatus* strains were detected. Six percent of the samples were positive for *Aspergillus* section *Fumigati* strains, all of which were azole-resistant. The strains were mainly *Aspergillus udagawae* (93.75%) and *Aspergillus lentulus* (6.25%). The predominant azole-resistant *Aspergillus* species were *Aspergillus* section *Nigri* strains, which were found in 75 soil samples. Conclusions: This study highlights the importance of understanding fungal resistance in vineyard soils for both the agricultural and clinical sectors. The presence of resistant strains may affect vine health and wine production while also constituting a challenge in the selection of effective treatments against severe and potentially fatal fungal infections in humans, stressing the importance of species-specific antifungal resistance knowledge.

## 1. Introduction

*Aspergillus fumigatus* is a species of fungus from the genus *Aspergillus*, which is commonly found in soil and decaying organic matter. The taxonomy of the genus *Aspergillus* is complex and has been divided into several sections based on morphological and physiological characteristics. Some of these sections include *Fumigati*, *Flavi*, *Nigri*, *Circumdati*, and *Terrei*, among others. Section *Fumigati* is a key group within this genus that contains several species, with *A. fumigatus* being the most prevalent and medically significant. Other species within the *Fumigati* section include *A. fischeri*, *A. lentulus*, *A. viridinutans*, *A. novofumigatus*, *A. udagawae*, and *A. felis*. While these species are less commonly encountered than *A. fumigatus*, they are noteworthy for their potential to impact agriculture and human health [1].

*Aspergillus* section *Fumigati* fungi are associated with substantial agricultural losses, especially in vineyards. They can lead to root rot, vascular wilt, and crown rot in a variety of plants, impacting both crop yield and quality. The fungal conidia are predominantly found in the air and soil, with conidial loads reaching up to 100 conidia/m^3^ of air [2]. In humans, the conidia of *Aspergillus* are easily inhaled and can lead to a wide range of diseases collectively known as aspergillosis. In healthy individuals, the immune system generally eliminates these inhaled spores, preventing disease. However, in immunocompromised individuals or those with chronic lung conditions, these conidia can germinate, leading to invasive aspergillosis—a serious, often deadly infection. Other health conditions linked to this fungal group include chronic pulmonary aspergillosis, allergic bronchopulmonary aspergillosis, and *Aspergillus* sinusitis. Data published in 2013 by the European Centre for Disease Prevention and Control (ECDC) estimated that aspergillosis affected an average of 2.3 million people per year in Europe, with 63,000 of these cases presenting the invasive form, which yields potentially life-threatening risks [3]. In Romania, there is no national monitoring system in place for fungal infections, but statistical data from the scientific literature suggest an incidence of at least 31,000 cases of aspergillosis per year [4]. In recent years, there has been an alarming rise in the number of triazole-resistant *A. fumigatus* strains within the *Fumigati* section. Triazoles are a group of antifungal agents commonly used to treat aspergillosis in humans and to control fungal diseases in agriculture. Resistance to these agents significantly complicates treatment and disease control efforts, making it a critical area of concern for both the public health and agricultural sectors.

In Romania, vineyards are a vital part of the nation’s agricultural fabric. They are spread across 189,000 hectares and encompass more than 250 wineries. With an annual wine production of approximately 4.5 million hectoliters as of 2021, Romania is recognized as the sixth-largest wine producer in Europe [5]. These vineyards not only significantly bolster the economy but also contribute profoundly to the cultural identity of the country, reflecting centuries of winemaking tradition and heritage. Despite its importance, there has been a surprising dearth of research into the prevalence of triazole-resistant *A. fumigatus* in Romanian vineyards. Given that this fungus can significantly affect the quality and yield of grapes, it is crucial to understand the scale and extent of this issue, particularly in the context of its potential impacts on both viticulture and public health [6].

The utilization of fungicides in Romanian vineyards is a common practice and is vital for maintaining crop health and productivity. Notably, the triazole group of fungicides, including tebuconazole, propiconazole, and difenoconazole, is used extensively. Collectively, Romanian vineyards use an estimated 15,000 metric tons of these fungicides annually to protect their vines against harmful fungal diseases such as powdery mildew and *Botrytis* rot [7]. However, this high level of fungicide use is not without consequences. The constant exposure of *A. fumigatus* to these fungicides is believed to be a primary driver of the development of triazole-resistant strains. Consequently, vineyards, where these fungicides are applied regularly and in significant quantities, could potentially function as significant hotspots for the proliferation of resistant strains.

Antifungal drugs based on triazole compounds (voriconazole, itraconazole, posaconazole, and isavuconazole) also currently represent the most efficient therapeutic option in terms of treatment success and cost [3]. Serial clinical case studies have shown that patients with invasive aspergillosis treated with voriconazole have a survival rate of 35%, compared to only 10% for those treated with amphotericin B, a drug that is not commercially available in Romania [8,9]. All of the above constitute strong reasons for determining the prevalence in the environment of *Aspergillus fumigatus* species resistant to azole compounds and for monitoring the risk factors that could influence their emergence.

The research presented in this study aimed to provide an estimate of the prevalence of triazole-resistant *Aspergillus* species from the section *Fumigati* in Romanian vineyards, elucidating the impact of agricultural practices on the development of fungal resistance and shedding light on potential risks to the viticulture industry and public health.

## 2. Results

### 2.1. Vineyards and Treatments Applied to Soil

In the present study, a total of seven vineyards, encompassing both privately owned and state-operated establishments, allowed the collection and analysis of soil samples. After securing the necessary approvals, a detailed questionnaire regarding the annual soil treatment protocols was extended to either the owner, the managerial representative, or an appropriately qualified individual associated with the vineyard. It should be noted that participation in the questionnaire was not mandatory. The data acquired regarding the soil treatments are presented in Table 1.

### 2.2. Screening of Azole-Resistant Fungi

Following the azole-resistance screening of soil samples from seven private and state-owned vineyards (Vineyards 1 through 7) in Romania, 109 of 265 soil samples were positive for *Aspergillus* spp. To avoid the inclusion bias of possible clonal colonies, when presented with multiple morphologically identical colonies on a single plate, only one colony from each plate was further analyzed. As a result, a total of 114 *Aspergillus* spp. isolates were retrieved. *Aspergillus* section *Nigri* emerged as the predominant *Aspergillus* species, with 75 isolates (65.79% of all *Aspergillus* isolates), followed by *Aspergillus* section *Fumigati* (16 isolates, 14.03% of all *Aspergillus* isolates), *Aspergillus* section *Usti* (13 isolates, 11.4%), *Aspergillus* section *Flavi* (8 isolates, 7.01%), and *Aspergillus* section *Terrei* (2 isolates, 1.75%).

A varied prevalence of resistance to itraconazole and voriconazole was observed among different fungal genera across the vineyards. Notably, *Aspergillus* section *Nigri* demonstrated a prevalence of itraconazole resistance ranging from as low as 7.5% (3 positive samples out of 40) in Vineyard 6 to as high as 40% (8 positive samples out of 20) in Vineyard 7. For voriconazole, the resistance in this section was generally lower, with no resistant isolates identified in Vineyard 6, while Vineyard 7 exhibited a prevalence of 15%. A noteworthy number of isolates presented cross-resistance between itraconazole and voriconazole (14 isolates; 18.6% of all *Aspergillus* section *Nigri* isolates).

*Aspergillus* section *Fumigati* showed notable resistance patterns. In Vineyard 5, the prevalence of resistance to both itraconazole and voriconazole reached 15%, indicating a significant challenge in controlling this section with these antifungals. Notably, all identified isolates presented cross-resistance to itraconazole and voriconazole.

For *Aspergillus* section *Usti*, resistance to both itraconazole and voriconazole was observed, peaking at a prevalence of 7.5% in Vineyard 4 for both antifungals. Cross-resistance was identified in seven isolates (53.8%).

*Aspergillus* section *Flavi* exhibited a moderate prevalence of itraconazole resistance, with the highest being 8% in Vineyard 3. Its resistance to voriconazole was lower, peaking at 4% in the same vineyard. Cross-resistance to azoles was identified in two isolates (25%).

*Aspergillus* section *Terrei* was found in only two samples, but both isolates presented cross-resistance to itraconazole and voriconazole (100%).

Mucorales were isolated from 98 (37%) samples and exhibited a unique resistance pattern, with a pronounced inclination towards voriconazole resistance and a moderate rate of azole cross-resistance (22 isolates, 22.45%). *Penicillium* spp. were found in 39 samples, with no recorded resistance to voriconazole.

The results are presented in detail in Table 2.

### 2.3. Azole Susceptibility Testing of Aspergillus Section Fumigati Isolates

The results presented in Table 3 show the susceptibility patterns for individual isolates of Aspergillus section Fumigati isolated from soil samples to itraconazole, voriconazole, and posaconazole. All isolated cryptic Aspergillus section Fumigati were non-wild-type. In Vineyard 2, we recovered six distinct isolates of Aspergillus udagawae from six separate soil samples. Although these isolates were of the same species and were collected from the same vineyard, each exhibited a unique MIC value, indicating individual variations in antifungal susceptibility. This pattern of variability in MIC values was consistent across the other vineyards as well. In Vineyard 3, two Aspergillus udagawae isolates from different soil samples exhibited distinct MIC values. Similarly, two isolates from Vineyard 4 and five isolates from Vineyard 5, each derived from separate soil samples, showed unique MIC values. This demonstrates the heterogeneity in antifungal resistance, even within the same species from the same vineyard, underscoring the importance of analyzing individual isolates to accurately assess resistance profiles.

## 3. Discussion

Understanding fungal species and their resistance patterns within vineyard soil environments is crucial for both the agricultural and medical sectors. In this investigation, we analyzed soil samples from various Romanian vineyards for fungi resistant to azole compounds, and our findings present both consistent and contrasting results relative to previous studies.

Following the screening of soil samples for fungi resistant to azoles, *Aspergillus* section *Nigri* emerged as the predominant *Aspergillus* species in the present study, as it was identified in 75 samples out of a total of 265. This was in accordance with previous research on species populations in vineyard soil [10,11]. Mucorales was the most frequently identified filamentous fungus (found in 98 samples), followed by *Penicillium* spp. (39 samples), *Aspergillus* section *Fumigati* (16 samples), *Aspergillus* section *Usti* (13 samples), and *Aspergillus* section *Flavi* (8 samples). *Aspergillus* section *Terrei* were found in only two samples.

*Aspergillus* section *Fumigati* isolates showed elevated MICs for both itraconazole and voriconazole, highlighting its potential threat in treatment scenarios. Our results indicate that vineyard soils in the studied Romanian regions are not currently hotspots of azole resistance in *Aspergillus fumigatus*. We did not detect any azole-resistant *A. fumigatus* strains while registering a 6.04% (16 out of 265 soil samples) prevalence of non-wild-type cryptic *Aspergillus* section *Fumigati* strains. The predominant non-wild-type species were *Aspergillus udagawae* (15; 93.75%) followed by *Aspergillus lentulus* (1; 6.25%).

These results contrast with the environmental resistance rates reported in Europe, which have shown considerable variation. Some studies indicate a resistance prevalence nearing 20%, while others report a minimal incidence of resistant environmental isolates [12,13,14,15,16,17]. Differences in outcomes might stem from methodological variations, such as the use of azole selection during the isolation process or the inclusion of potentially clonal isolates from specific samples, which can skew results. For our part, we focused on one isolate per soil sample to minimize the potential bias from clonal isolates.

Another notable aspect is the differentiation between “environmental” isolates from rural or agricultural settings and urban areas. A recent study underscored this by revealing that rural regions in the UK had markedly lower resistance rates (1.1%) compared to urban locales (13.8%) [16]. It is pertinent to mention that the vineyards in our study were exclusively located in rural regions.

Historically, much of the research emphasis has been on *A. fumigatus sensu stricto*, often overlooking cryptic species from the *Fumigati* section. However, surveillance reports from medical units have indicated rising incidences of infections with cryptic *Aspergillus*. A study detailing the speciation and antifungal resistance profiles of *Aspergillus* spp. that cause invasive fungal diseases in Queensland, Australia, identified the most commonly encountered species as *Aspergillus* section *Fumigati*. Cryptic *Aspergillus* spp. had a prevalence of 6.4% [18]. A separate study on the species distribution and antifungal susceptibilities of *Aspergillus* section *Fumigati* isolates from clinical samples in the US observed a 4% infection incidence with cryptic *Aspergillus* from the *Fumigati* section [19]. In the 6-year, prospective TRANS-NET surveillance study focused on solid-organ transplant recipients and those undergoing hematopoietic stem cell transplantation in the US and Canada, Balajee et al. discovered that 93.9% of infections were caused by *A. fumigatus sensu stricto*. The remaining 6.1% were other species, including *A. lentulus* (2.7%) and *A. udagawae* (2.0%) [20]. Similarly, a population-based survey in Spain found that while cryptic species constituted up to 12% of all *Aspergillus* species, 3.7% within the *Fumigati* section were not *A. fumigatus sensu stricto* [21]. The heightened resistance of these cryptic species, despite their lower prevalence compared to *A. fumigatus sensu stricto*, necessitates their consideration in future studies.

The *Aspergillus* spp. prevalence in Romania, in both clinical and environmental settings, remains under-researched. During the COVID-19 pandemic, a marked increase in fungal infections was observed. A decade-long observational study at Târgu Mureș Hospital, Romania, identified *Aspergillus* section *Flavi* (58.82%) as the predominant invasive mold, followed by *Aspergillus* section *Nigri* (20.59%), albeit with an overall low incidence rate of 0.40%. Notably, there was a significant uptick in positive fungal cases in 2021, and mold infections were linked to increased COVID-19-associated mortality [22]. Subsequent studies spanning six pandemic waves highlighted variable fungal species prevalence. *Aspergillus* spp. were most prominent in the third wave, whereas Mucorales peaked in the first. Among *Aspergillus* infections, drug resistance patterns varied, with a minority showing resistance to three or more antifungals [23].

The current study observed different antifungal MIC patterns of mainly *A. udagawae* species across various vineyards, with a single instance of *A. lentulus* in Vineyard 5. The samples from Vineyard 2 displayed considerable variability in their MICs for itraconazole, ranging from 2 to >8 mg/L. Similarly, the voriconazole MICs were also evidently elevated, with a significant proportion showing levels of >8 mg/L. Most isolates presented posaconazole MIC values centered around 4 mg/L or less. In Vineyard 3, while one sample exhibited high MICs for both itraconazole and voriconazole (>8 mg/L), the other showed considerably lower values, hinting at potential variability even within the same vineyard. The Vineyard 4 samples displayed a moderate range of MICs across all three antifungals. Vineyard 5, however, stood out not just because of the presence of *A. lentulus* but also due to the broader range of MIC patterns among the *A. udagawae* samples. The data pointed to both high (>8 mg/L) and relatively low (2–4 mg/L) MICs for itraconazole and voriconazole. Likewise, posaconazole MICs varied considerably, with values ranging from 0.5 to >4 mg/L.

In the present study, we observed the concurrent growth of Mucorales and *Penicillium* spp. alongside *Aspergillus* species in our soil samples, a phenomenon warranting further discussion. The co-occurrence of these two genera in environmental samples has also been noted in other studies focusing on resistant *Aspergillus* spp. [24,25]. The presence of multiple fungal species on the same culture plates suggests the potential for competitive interference, which could significantly impact the recovery and isolation of *Aspergillus* spp. colonies. Mucorales and *Penicillium* spp., known for their rapid growth and expansive mycelial networks, may outcompete *Aspergillus* spp. for space and nutrients on culture media [26]. This competitive environment could have led to an underestimation of *Aspergillus* spp. isolates in our soil samples.

Consideration must be given to the fungicide treatments used in the vineyards included in the present study, as these practices directly influence the fungal populations and their resistance patterns. Contact fungicides such as calcium polysulfide solution, Kumulus WDG, and Folpan 80 WG were used extensively across Vineyards 2, 3, and 7. This type of fungicide is applied primarily for controlling powdery mildew and downy mildew. Its mode of action is preventive, requiring application to the plant surfaces before infection occurs. These fungicides are not absorbed by the plant and remain on the surface, necessitating repeated applications, especially after rain or irrigation [27]. Despite their potential phytotoxicity, their advantage lies in leaving no residual chemicals on produce, making them ideal for use close to harvest [28].

Systemic fungicides such as Profiler, Forum Gold, and Ridomil were also heavily employed in Vineyards 2, 3, and 7. They are absorbed by the plant and combat fungal infections from within. They are effective against diseases like downy and powdery mildew, gray mold, and *Aspergillus* spp. Systemic fungicides can eradicate or suppress fungal growth after infection, providing a longer-lasting protective effect. However, they carry a higher risk of fungi developing resistance, necessitating careful management and rotation with different classes of fungicides [29]. Some fungicides, such as Forum Gold and Melody Compact, provide both systemic and contact actions, offering a more comprehensive approach to fungal control. Contact fungicides offer short-term, surface-level protection suitable for pre-harvest periods, while systemic fungicides provide deeper, longer-lasting protection but with considerations for resistance management. The strategic use of both types in rotation, as seen in these vineyards, has been shown to help effectively manage fungal diseases while minimizing the risk of resistance development [29].

A significant study limitation arose from the unavailability of complete data concerning crop treatment practices. Such data, particularly the amount of fungicide applied and the dates of treatments, are notoriously difficult to obtain. For this study, complete treatment details were only available for Vineyards 2 and 3. This limited the availability of treatment data and hampered our ability to draw concrete correlations between soil treatments and the observed resistance patterns. In essence, while *A. udagawae* showed elevated and varied MIC patterns across vineyards and azoles, without comprehensive treatment data, the links between fungicide use and the occurrence of non-wild-type cryptic *Aspergillus* section *Fumigati* remain speculative.

Nevertheless, the findings from this investigation hold substantial significance. From an agrarian standpoint, these fungi’s presence and resistance patterns can critically impact vine health, yield, and the overall wine production process. On the medical front, these non-wild-type strains, if they contaminate grape products or become airborne, present substantial clinical threats, especially for immunocompromised individuals. The elevated MICs observed for common antifungals mean treatment options may be limited and potentially less effective. Given the prominence of vineyards in Romania’s agrarian landscape, understanding these resistance patterns and their implications is becoming vitally important for both the agricultural and clinical domains. Therefore, this study underscores the need for species-specific antifungal resistance knowledge, heralding a direction for more targeted and efficient interventions.

## 4. Materials and Methods

### 4.1. Soil Sample Collection

**Figure 1:**
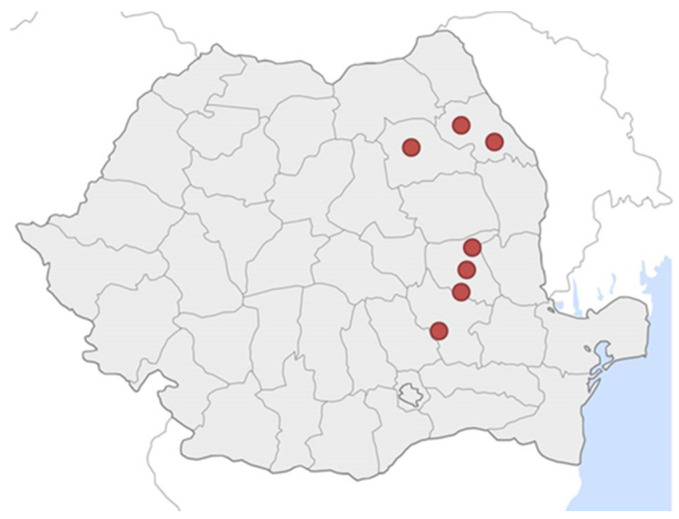
Graphical representation of the wine regions from which soil samples were collected. The red dots represent the collection locations within Romania.

From August to September 2019, 265 soil samples were collected from private and state-operated vineyards (Research and Development Stations for Viticulture and Vinification) in 7 different regions of Romania (Figure 1). All vineyards were located in rural locations. Written permission was obtained from the owner/manager of the vineyard for access to the property, soil sampling, and the publication of the data obtained from the analysis of the samples. Additional information regarding the general management of the vineyard (soil type, type of fertilizer used, natural pest treatment scheme, types of fungicides and pesticides used, and treatment frequency) was sought from the owner/manager of the vineyard or qualified personnel through a questionnaire. Participation in the present study was voluntary, and as per the written permission, data regarding the names and particularities of the vineyards were kept anonymous. The questionnaire was written in Romanian. In Supplementary Figure S1, the original version of the questionnaire is presented, as well as the English translation.

**Figure 2:**
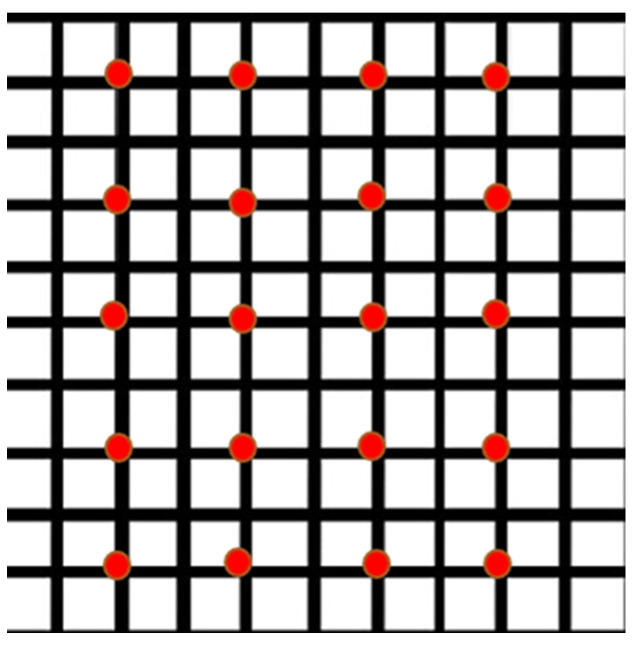
Graphical representation of the soil sampling system on a 10-hectare vineyard, following the grapevine row. The red dots represent the sampling locations.

The number of soil samples collected from each vineyard was directly proportional to the size of the property, where 20 samples were collected for every 10 hectares of land. As a result, the sample size per vineyard ranged from 20 to 55. The collection was carried out systematically following a grid pattern, from equidistant locations, with the avoidance of vine rows located at the ends of the properties and those located in the immediate vicinity of public roads (Figure 2).

Sampling occurred on dry, sunny days (temperatures between 30 and 35 °C). At each location, approximately 10 g of dry surface soil was collected in a sterile container (sterile urine container) using a sterile plastic spoon. A new sterile spoon was used for each sample. The samples were labeled and transported in a sealed package to the Microbiology Division of the Iuliu Hațieganu University of Medicine and Pharmacy in Cluj-Napoca, Romania, for processing. The samples were stored in sterile, tightly closed containers at room temperature and were protected from direct sunlight.

### 4.2. Soil Sample Processing

The isolation and preliminary screening of *Aspergillus* section *Fumigati* species resistant to triazole compounds used 2 g of soil from each collected sample. The 2 g were dissolved in a solution composed of 8 mL of sterile distilled water, 1% Tween 20 (Thermo Fisher Scientific Inc., Waltham, MA, USA), and 0.5 g/L chloramphenicol (Thermo Fisher Scientific Inc., Waltham, MA, USA). The sample was vortexed at a high speed for 1 min and left to settle for 60 min at room temperature. Using an automatic pipette, 100 µL of the supernatant was taken and then inoculated onto 3 media: a Sabouraud medium (Oxoid, Basingstoke, United Kingdom) supplemented with 4 mg/L itraconazole (Acros Organics, Antwerp, Belgium), a Sabouraud medium supplemented with 2 mg/L voriconazole (Thermo Fisher Scientific Inc., Waltham, MA, USA), and an unsupplemented Sabouraud medium (control plate). The antifungal-supplemented media were prepared in the Microbiology Division laboratory. To prepare the plates supplemented with antifungals, the required amounts of the antifungals (4 mg of itraconazole or 2 mg of voriconazole) were dissolved in 1 mL of DMSO and added to 1 L of Sabouraud medium supplemented with chloramphenicol. Once inoculated, the plates were incubated with the thermostat at 35 °C and examined after 48 and 72 h [30].

The preliminary identification of the fungi that grew on the antifungal-supplemented media, as well as an equal number of isolates from the control plates, was performed macroscopically by analyzing the morphology of the fungal colonies (shape, size, surface, and color) using a Zeiss stereo microscope (Carl Zeiss MicroImaging GmbH, Oberkochen, Germany). The colonies were then analyzed under a light microscope using the scotch technique. A piece of transparent scotch tape (1 cm in length and 0.5 cm in width) was taped onto the surface of the fungal colony so that the fungal heads adhered to the scotch tape. The tape was then placed on a microscope slide onto which a drop of lactophenol blue was applied. The microscope slide was then viewed under the light microscope (10x objective followed by 40x objective). The identification of *Aspergillus* section *Fumigati* isolates based on the macroscopic and microscopic morphology was confirmed by a specialist in clinical microbiology. The numbers and types of filamentous fungi isolated from each soil sample were recorded. In order to avoid the inclusion bias of possible clonal colonies, when presented with multiple morphologically identical colonies on a single plate, only one colony from each plate was further analyzed. For the preservation of isolates identified as *Aspergillus* section *Fumigati*, spores and fungal heads were collected and suspended in an Eppendorf tube with 1.5 mL of 10% glycerol [31] and stored at −20 °C. The isolates were kept until species identification was performed via molecular analysis.

### 4.3. Molecular Analysis

The species identification of the isolates was performed through molecular analysis of the fungal genome. For this, samples of azole-resistant isolates were sent to the Strasbourg University Hospital laboratory (France). The sample preparation for DNA extraction consisted of inoculating each isolate in 2 mL of Sabouraud medium, followed by incubation for 72 h at 37 °C. The DNA of the azole-resistant isolates was extracted using the MagNAPure 96 System (F. Hoffmann-La Roche AG, Basel, Switzerland) automated extractor. To confirm the identification of each *Aspergillus* isolate at the species level, the Beta-tubulin and Calmodulin genes were amplified using the PCR technique. Primers used for amplification are presented in Table 4. The amplified genes were then purified and sent for sequencing by Eurofins Genomics (Ebersberg, Germany GmbH).

### 4.4. Azole Susceptibility Testing

The susceptibility of *Aspergillus* section *Fumigati* isolates to itraconazole, voriconazole, and posaconazole was tested using the microdilution method (EUCAST) [32]. *Candida parapsilosis* ATCC 22019 and *Candida krusei* ATCC 6258 were used for quality control for each performed test. According to the EUCAST breakpoints (www.eucast.org; accessed on 29 November 2023), isolates that exhibited minimum inhibitory concentrations (MICs) of itraconazole and voriconazole greater than 1 mg/L and those with a posaconazole MIC greater than 0.25 mg/L were considered resistant. *Aspergillus* section *Fumigati* with itraconazole and voriconazole MICs less than or equal to 1 mg/L and those with posaconazole MICs less than or equal to 0.125 mg/L were considered susceptible. To increase the accuracy of the results and lower the possibility of pipetting errors, all MIC determinations were performed 4 times for each *Aspergillus* section *Fumigati* isolate (as seen in Supplementary Figure S2).

## 5. Conclusions

In conclusion, our targeted exploration of *Aspergillus* section *Fumigati* in Romanian vineyards provides illuminating findings. Notably, despite the broader concerns surrounding azole resistance, we did not detect any strains of *A. fumigatus* exhibiting resistance to triazoles. This discovery offers reassurance regarding the state of azole resistance in this specific section within our study region. Nevertheless, the presence of cryptic species within the *Fumigati* section underscores the evolving complexity of fungal ecosystems and the importance of continuous surveillance. Our study emphasizes the need for nuanced understanding and monitoring of specific fungal species, as their resistance profiles can hold significant implications for both agricultural practices and clinical interventions in Romania.

## Figures and Tables

**Table 1:** Treatment protocols utilized by vineyards from which samples were collected.

Order of Treatments	Product/Manufacturer	Type	Azole Component	Dosage	Mode of Action	Target Diseases and Pests
	Vineyard 2					
1	Calcium polysulfide solution	Fungicide, Insecticide, Acaricide	No	12 L/ha	Contact	Powdery mildew, Moths, Mites
2	Profiler (Bayer, Leverkusen, Germany)	Fungicide	No	2.5 kg/ha	Systemic	Downy mildew
	Topas (Syngenta, Basel, Switzerland)	Fungicide	Penconazole	0.25 L/ha	Contact	Powdery mildew
	Envidor (Bayer, Leverkusen, Germany)	Acaricide	No	0.4 L/ha	Contact	Erineum mites
3	Forum Gold (BASF, Ludwigshafen, Germany)	Fungicide	No	1.56 kg/ha	Systemic contact	Downy mildew
	Vivando (BASF, Ludwigshafen, Germany)	Fungicide	No	0.2 L/ha	Systemic contact	Powdery mildew
	Envidor	Acaricide	No	0.4 L/ha	Contact	Erineum mites
4	Forum Gold	Fungicide	No	1.56 kg/ha	Systemic contact	Downy mildew
	Vivando	Fungicide	No	0.2 L/ha	Systemic contact	Powdery mildew
5	Folpan 80 WG (Adama, Ashdod, Israel)	Fungicide	No	1.5 kg/ha	Contact	Downy mildew, Gray mold
	Kumulus WDG (BASF, Ludwigshafen, Germany)	Fungicide	No	3.0 kg/ha	Contact	Powdery mildew
6	Ridomil (Syngenta, Basel, Switzerland)	Fungicide	No	2.5 kg/ha	Systemic	Downy mildew
	Kumulus WDG	Fungicide	No	3.0 kg/ha	Contact	Powdery mildew
	Cantus^®^ (BASF, Ludwigshafen, Germany)	Fungicide	No	1.2 kg/ha	Systemic	Gray mold
7	Ridomil	Fungicide	No	2.5 kg/ha	Systemic	Downy mildew
	Kumulus WDG	Fungicide	No	3.0 kg/ha	Contact	Powdery mildew
	Cantus^®^	Fungicide	No	1.2 kg/ha	Systemic	Gray mold
8	Bouillie bordelaise	Fungicide	No	5.0 kg/ha	Contact	Downy mildew
	Vineyard 3					
1	Novozir MN 80 (Belchim, Bucuresti, Romania)	Fungicide	No	2.0 kg/ha	Contact	Downy mildew
	Polisulfură de calciu WDG	Fungicide	No	3.0 kg/ha	Contact	Powdery mildew
2	Manconova (Trustchem Co. Ltd., Hangzhou, China)	Fungicide	No	2.5 kg/ha	Contact	Downy mildew
	Kumulus WDG	Fungicide	No	3.0 kg/ha	Contact	Powdery mildew
	Envidor	Acaricide	No	0.4 L/ha	Contact	Erineum mites
3	Forum Gold	Fungicide	No	1.56 kg/ha	Systemic contact	Downy mildew
	Karathane^TM^ Gold 350 EC (Corteva^TM^ Agriscience, Indianapolis, Indiana, United States)	Fungicide	No	0.5 L/ha	Systemic	Powdery mildew
4	Curzate F (Corteva^TM^ Agriscience, Indianapolis, Indiana, United States)	Fungicide	No	2.5 L/ha	Systemic	Downy mildew
	Luna Experience 400 SC (Bayer, Leverkusen, Germany)	Fungicide	Tebuconazole	0.5 L/ha	Systemic	Powdery mildew
5	Verita (Bayer, Leverkusen, Germany)	Fungicide	No	2.5 kg/ha	Systemic	Downy mildew
	Falcon 460 EC (Bayer, Leverkusen, Germany)	Fungicide	No	0.3 L/ha	Systemic	Powdery mildew
	Sulfomat (Mifalchim, Onești, Romania)	Fungicide	No	4 kg/ha	Contact	Powdery mildew
	Pyrus 400 SC (Agriphar, Ougree, Belarus)	Fungicide	No	1.5 L/ha	Systemic	Gray mold
6	Valis M (Belchim, Bucuresti, Romania)	Fungicide	No	2.0 kg/ha	Systemic contact	Downy mildew, Gray mold
	Folpan	Fungicide	No	1.5 kg/ha	Contact	Downy mildew, Gray mold
	Kumulus WDG	Fungicide	No	3.0 kg/ha	Contact	Powdery mildew
	Bumper 250 EC (Adama, Ashdod, Israel)	Fungicide	Propiconazole	0.2 L/ha	Systemic	Powdery mildew
7	Pergado F (Syngenta, Basel, Switzerland)	Fungicide	No	2.5 kg/ha	Systemic contact	Downy mildew
	Vivando	Fungicide	No	0.2 L/ha	Systemic contact	Powdery mildew
	Karathane^TM^ Gold 350 EC	Fungicide	No	0.5 L/ha	Systemic	Powdery mildew
8	Melody Compact (Bayer, Leverkusen, Germany)	Fungicide	No	1.5 kg/ha	Systemic contact	Downy mildew
	Sulfomat	Fungicide	No	4 kg/ha	Contact	Powdery mildew
	Kumulus WDG	Fungicide	No	3.0 kg/ha	Contact	Powdery mildew
	Pyrinex (Adama, Ashdod, Israel)	Insecticide	No	2.2 L/ha	Contact ingestion	Moths
	Vineyard 1, 4–6—Information not provided					
	Vineyard 7					
6–8/year	Switch 62.5 WG (Syngenta, Basel, Switzerland)	Fungicide	No	1 kg/ha	Systemic contact	Gray mold, *Aspergillus* spp.
	NPK 16-16-16	Fertilizer	No			
	Other					Downy mildew, Powdery mildew

The treatments were administered over the duration of 1 year (2019, year of soil sampling) and are presented in the order of administration. The treatments in Vineyard 7 consisted of 6–8 (depending on year) administrations of the presented products, without mention of specific times or an order of administration.

**Table 2:** Soil screening results: zonal distribution of itraconazole- and voriconazole-resistant fungi.

Vineyard	No. of Samples Collected per Vineyard	Itraconazole-Resistant Fungi (No. of Positive Samples; Percentage)	Voriconazole-Resistant Fungi (No. of Positive Samples; Percentage)
1	20	*Aspergillus* section *Nigri* (5; 25%)	*Aspergillus* section *Nigri* (2; 10%)
		*Aspergillus* section *Usti* (1; 5%)	*Aspergillus* section *Usti* (1; 5%)
		*Penicillium* spp. (7; 35%)	*-*
		Mucorales (2; 10%)	Mucorales (8; 40%)
2	55	*Aspergillus* section *Nigri* (13; 23.64%)	*Aspergillus* section *Nigri* (3; 5.45%)
		*Aspergillus* section *Flavi* (2; 3.64%)	-
		*Aspergillus* section *Usti* (3; 5.45%)	-
		*Aspergillus* section *Fumigati* (6; 10.9%)	*Aspergillus* section *Fumigati* (6; 10.9%)
		Mucorales (8; 14.55%)	Mucorales (15; 27.27%)
3	50	*Aspergillus* section *Nigri* (11; 22%)	*Aspergillus* section *Nigri* (7; 14%)
		*Aspergillus* section *Flavi* (4; 8%)	*Aspergillus* section *Flavi* (2; 4%)
		*Aspergillus* section *Usti* (3; 6%)	-
		*Aspergillus* section *Fumigati* (2; 4%)	*Aspergillus* section *Fumigati* (2; 4%)
		*Aspergillus* section *Terrei* (2; 4%)	*Aspergillus* section *Terrei* (2; 4%)
		*Penicillium* spp. (6; 12%)	-
		Mucorales (7; 14%)	Mucorales (16; 32%)
4	40	*Aspergillus* section *Nigri* (11; 27.5%)	*Aspergillus* section *Nigri* (4; 10%)
		*Aspergillus* section *Usti* (3; 7.5%)	*Aspergillus* section *Usti* (3; 7.5%)
		*Aspergillus* section *Fumigati* (2; 5%)	*Aspergillus* section *Fumigati* (2; 5%)
		*Penicillium* spp. (6; 15%)	-
		Mucorales (3; 7.5%)	Mucorales (15; 37.5%)
5	40	*Aspergillus* section *Nigri* (14; 35%)	*Aspergillus* section *Nigri* (5; 12.5%)
		*Aspergillus* section *Flavi* (2; 5%)	-
		*Aspergillus* section *Usti* (2; 5%)	*Aspergillus* section *Usti* (2; 5%)
		*Aspergillus* section *Fumigati* (6; 15%)	*Aspergillus* section *Fumigati* (6; 15%)
		*Penicillium* spp. (5; 12.5%)	-
		Mucorales (7; 17.5%)	Mucorales (15; 37.5%)
6	40	*Aspergillus* section *Nigri* (3; 7.5%)	-
		*Penicillium* spp. (9; 22.5%)	-
		Mucorales (1; 2.5%)	Mucorales (15; 37.5%)
7	20	*Aspergillus* section *Nigri* (8; 40%)	*Aspergillus* section *Nigri* (3; 15%)
		*Aspergillus* section *Usti* (1; 5%)	*Aspergillus* section *Usti* (1; 5%)
		*Penicillium* spp. (6; 30%)	-
		Mucorales (1; 5%)	Mucorales (8; 40%)

**Table 3:** Susceptibility of fungal isolates of interest to azole compounds (*Aspergillus* section *Fumigati*).

Vineyard	Species	Itraconazole	Voriconazole	Posaconazole
2	*Aspergillus udagawae*	8	>8	4
2	*Aspergillus udagawae*	>8	>8	>4
2	*Aspergillus udagawae*	8	>8	>4
2	*Aspergillus udagawae*	4	8	4
2	*Aspergillus udagawae*	8	4	2
2	*Aspergillus udagawae*	2	4	0.5
3	*Aspergillus udagawae*	2	1	0.5
3	*Aspergillus udagawae*	>8	>8	2
4	*Aspergillus udagawae*	2	4	0.5
4	*Aspergillus udagawae*	4	8	4
5	*Aspergillus udagawae*	8	4	4
5	*Aspergillus udagawae*	2	4	0.5
5	*Aspergillus lentulus*	8	4	4
5	*Aspergillus udagawae*	4	4	>4
5	*Aspergillus udagawae*	8	4	>4
5	*Aspergillus udagawae*	>8	>8	>4

Results are presented as minimum inhibitory concentrations (mg/L).

**Table 4:** Primers used for amplification of Calmodulin and Tubulin genes.

**Calmodulin**	CL1	20 pb	GAR TWC AAG GAG GCC TTC TC GARTWCAAGGAGGCCTTCTC
	CL2	21 pb	TTT TTG CAT CAT GAG TTG GAC TTTTTGCATCATGAGTTGGAC
**Tubulin**	Bt2A	24 pb	GGT AAC CAA ATC GGT GCT GCT TTC GGTAACCAAATCGGTGCTGCTTTC
	Bt2B	24 pb	ACC CTC AGT GTA GTG ACC CTT GGC ACCCTCAGTGTAGTGACCCTTGGC

## Data Availability

The data are contained within the article and the Supplementary Materials.

## Supplementary Materials

The following supporting information can be downloaded at https://www.mdpi.com/article/10.3390/antibiotics12121695/s1, Figure S1: Soil sampling agreement form and questionnaire; Figure S2: Microdilutions method for azole Minimum Inhibitory Concentration determination.
