# Supplementary material for: Prevalence of Azole-Resistant Aspergillus Section Fumigati Strains Isolated from Romanian Vineyard Soil Samples
Source: Antibiotics (Basel). 2023 Dec 3;12(12):1695. doi: 10.3390/antibiotics12121695 (PMC10741105; doi:10.3390/antibiotics12121695)
Supplement: Supplementary file 1 [file antibiotics-12-01695-s001.zip › Supplementary Figure S1.pdf]

U.M.F. "Iuliu Hatieganu" Cluj-Napoca

Disciplina de Microbiologie

UMF UNIVERSITATEA DE MEDICINĂ ŞI FARMACIE IULIU HAŢIEGANU CLUJ-NAPOCA

ACORD DE ACCES PE PROPRIETATE PENTRU PRELEVARE SOL

Nume Proprietar/Manager ____

Adresa Proprietatii ____

Strada Oras Judet

Telefon ___________________________

E-mail ____

In calitate de **proprietar/manager** al proprietatii corespunzatoare adresei mentionate anterior, permit reprezentantilor Disciplinei de Microbiologie din cadrul Universitatii de Medicina si Farmacie "Iuliu Hatieganu" intrarea pe proprietate pentru prelevarea mostrelor de sol cu scopul analizei si identificarii tulpinilor de *Aspergillus* (fungi).

Mi s-a explicat si inteleg pe deplin ca toate datele obtinute din analiza mostrelor de sol vor fi folosite doar in scop de cercertare, cu intentia de publicare în reviste stiintifice. Deasemenea, inteleg ca toate informatiile cu caracter personal (nume, adresa, numar de telefon etc.), precum si cele ce apartin proprietatii in cauza (nume, locatie, etc) nu constituie obiectul studiului si nu vor fi publicate de catre UMF "Iuliu Hatieganu" si reprezentantii sai. Am fost informat ca pot beneficia de accesul la datele obtinute in urma anlizei mostrelor de sol recoltate de pe proprietatea in cauza.

☐ Doresc sa primesc date obtinute in urma procesarii mostrelor

____

Semnatura Proprietar/Manager Data

Introducere

Speciile de *Aspergillus* sunt ciuperci saprofite ubiquitare, care se găsesc în numeroase nişe ecologice din întreaga lume. *Aspergillus fumigatus* este în mare parte responsabil pentru incidenţa crescută a aspergilozei la pacienţii imunocompromiţi. Azolii sunt medicamentele de prima linie utilizate prentru tratarea bolilor cauzate de *Aspergillus*, atât în mediul medical cât şi în cel agricol. Creşterea rezistenţei acestui fung la azoli a fost raportată global, fapt ce ameninţă eficacitatea antifungicelor pe bază de azoli utilizate în tratamentul infecţiilor umane.

Obiective

Studiul prezent urmăreşte să descopere prezenţa în sol de specii de *Aspergillus* rezistente la azoli.

Desfăşurarea studiului

În perioada 1 august – 30 octombrie 2019 se vor preleva probe de sol de suprafaţă din diverse zone viticole din România. Din fiecare arie viticolă se vor recolta 20 de mostre de sol (5 grame per mostră), acestea urmând să fie analizate în cadrul laboratorului Disciplinei de Microbiologie al UMF "Iuliu Haţieganu" Cluj-Napoca cu scopul izolării de fungi din genul Aspergillus.

*Pentru o mai bună documentare a studiului, vă rugăm să completaţi chestionarul de mai jos cu privire la caracteristicile solului ce urmează să fie analizat.

Tip sol	
Îngrăşământ folosit	
Dăunători naturali	
Fungicid folosit	
Alte pesticide	
Nr. utilizări per sezon	

Semnătura Proprietar/Manager Data

U.M.F. "Iuliu Hatieganu" Cluj-Napoca

Department of Microbiology

AGREEMENT FOR PROPERTY ACCESS FOR SOIL SAMPLING

Name Owner/Manager____

Property Address____

Street City County

Telephone ___________________________

E-mail ____

As the owner/manager of the property corresponding to the previously mentioned address, I allow representatives of the Microbiology Discipline from the University of Medicine and Pharmacy "Iuliu Hatieganu" to enter the property to collect soil samples for the purpose of analyzing and identifying strains of *Aspergillus* (fungi).

It has been explained to me and I fully understand that all data obtained from the analysis of the soil samples will only be used for research purposes, with the intention of publishing in scientific journals. Furthermore, I understand that all personal information (name, address, phone number, etc.), as well as information related to the property in question (name, location, etc.) are not the subject of the study and will not be published by UMF "Iuliu Hatieganu" and its representatives. I have been informed that I can have access to the data obtained from the analysis of the soil samples collected from the mentioned property.

☐ I wish to receive the data obtained after processing the samples.

____

Signature of Owner/Manager Date

Introduction

Aspergillus species are ubiquitous saprophytic fungi found in numerous ecological niches around the world. *Aspergillus fumigatus* is largely responsible for the increased incidence of aspergillosis in immunocompromised patients. Azoles are the first-line drugs used to treat diseases caused by *Aspergillus*, both in the medical and agricultural environment. The increasing resistance of this fungus to azoles has been reported globally, threatening the effectiveness of azole-based antifungals used in the treatment of human infections.

Objectives

The present study aims to discover the presence in soil of azole-resistant *Aspergillus* species.

Conduct of the Study

From August 1 to October 30, 2019, surface soil samples will be taken from various wine-growing areas in Romania. From each wine-growing area, 20 soil samples (5 grams per sample) will be collected, which will be analyzed at the Microbiology Discipline Laboratory of UMF "Iuliu Haţieganu" Cluj-Napoca with the purpose of isolating fungi from the Aspergillus genus.

*For better documentation of the study, please fill out the questionnaire below regarding the characteristics of the soil to be analyzed.

Soil type	
Fertilizer used	
Natural pests	
Fungicides used	
Other pesticides used	
No. of treatments per year	

Signature of Owner/Manager Date
