# Supplementary material for: Prevalence of Azole-Resistant Aspergillus Section Fumigati Strains Isolated from Romanian Vineyard Soil Samples
Source: Antibiotics (Basel). 2023 Dec 3;12(12):1695. doi: 10.3390/antibiotics12121695 (PMC10741105; doi:10.3390/antibiotics12121695)

Supplementary Figure S2. Serial microdilution on 96 well plate for 2 *Aspergillus* isolates. Determination of Minimum Inhibitory concentrations were conducted in quadruplicate for all isolates.

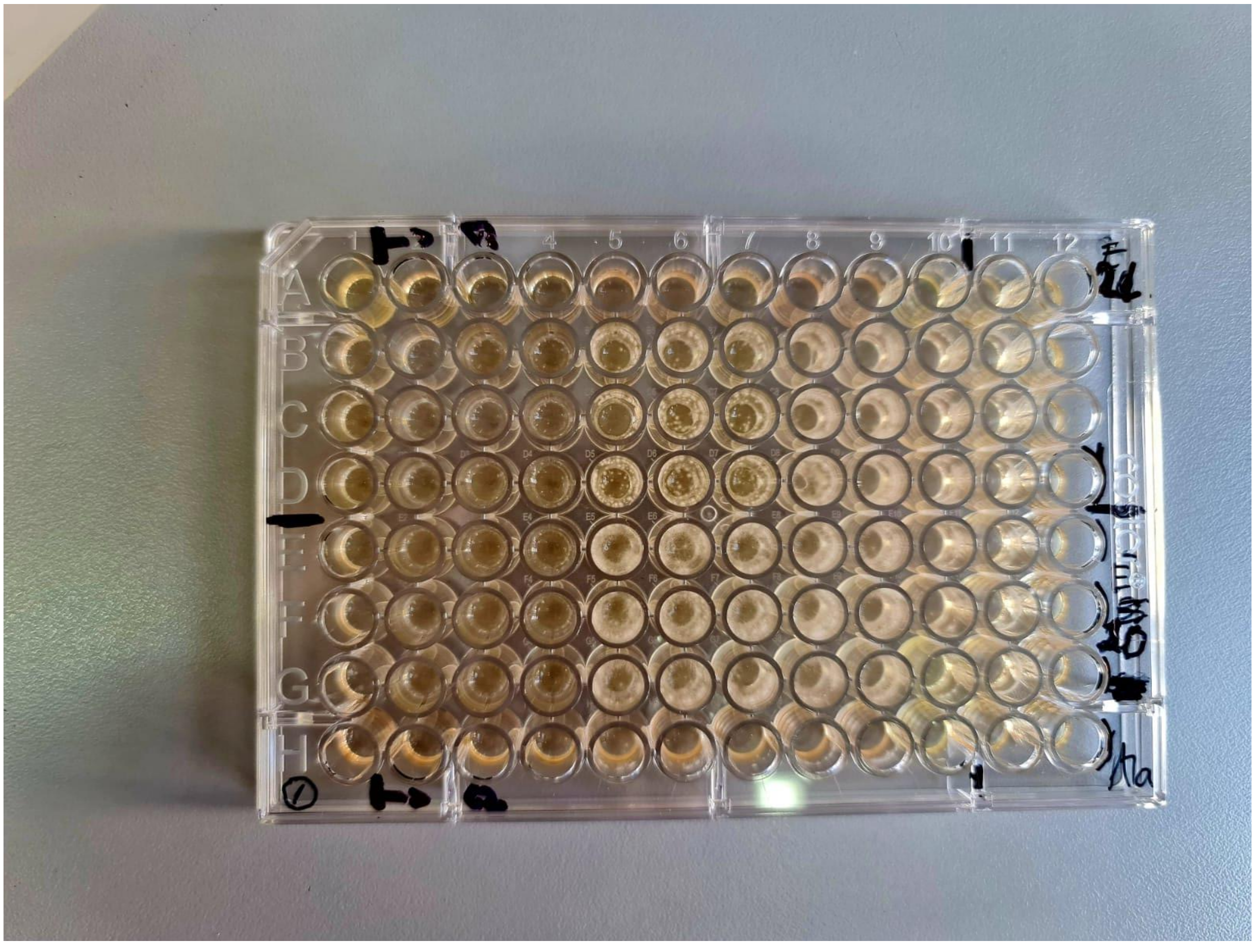

Supplement: Supplementary file 1 [file antibiotics-12-01695-s001.zip › Supplementary Figure S2.pdf]
